# Who participates in LDCT screening for the early detection of lung cancer after long-term occupational exposure to asbestos dust? Results of the analysis of administrative and medical routine data from the German Social Accident Insurance

**DOI:** 10.1186/s12890-026-04780-4

**Published:** 2026-10-12

**Authors:** Nikolas Oubaid, Felix Greiner, Helena Keller, Martin Ansgar Horn, Alexandra Marita Preisser, Volker Harth

**Affiliations:** Institute for Occupational and Maritime Medicine (ZfAM), University Medical Center Hamburg-Eppendorf, Hamburg, Germany

**Keywords:** Lung cancer screening, Early detection, Low-dose computed tomography (LDCT), Asbestos dust exposure, Occupational medicine, Smoking history, Patient participation, Secondary data analysis, Regression

## Abstract

**Background:**

Lung cancer is one of the most common cancers in Germany and is predominantly detected at an advanced stage. To ensure early detection, the German Social Accident Insurance (DGUV) offers lung cancer screening including a low-dose computed tomography (LDCT) for individuals with long-term occupational exposure to asbestos dust and a smoking history of at least 30 pack years. However, evidence remains scarce about what encourages this group of people to participate in LDCT screening after medical consultation.

**Methods:**

A secondary data analysis using routinely collected medical and administrative data between 2014 and 2024 was conducted in a cross-sectional design. This study aimed to investigate factors that could be used to predict the uptake of LDCT screening following a medical consultation. Exploratory logistic regression analysis was used to identify and assess possible predictors.

**Results:**

The sample included *n* = 14,491 individuals (97% male; average age 67 years; average asbestos dust exposure 21 years; average cigarette consumption 43 pack years). 74% of individuals underwent LDCT screening after medical consultation. 13 predictors were included in the regression model. The model demonstrated a low level of variance explanation (Nagelkerkes R^2^ = 0.026). Younger individuals (OR: 0.96, *p* < 0.001), individuals with prolonged exposure to asbestos dust (OR: 1.01, *p* = 0.011) and with a history of pneumonia (OR: 1.16, *p* = 0.011) were more likely to undergo LDCT screening.

**Conclusion:**

The predictors showed only a low explanatory power. Further research should include non-participants and expand the exploratory model to include theoretically grounded predictors.

**Supplementary Information:**

The online version contains supplementary material available at https://doi.org/10.1186/s12890-026-04780-4.

## Introduction

### Background

In terms of prevalence and mortality, lung cancer is one of the most prevalent and unfavorable cancers [1, 2]. It accounts for over two million diagnoses worldwide per year [2] where of 56,690 people being diagnosed in Germany in 2020 [1].

In scientific literature various factors have been described that increase lung cancer risk. The most significant of these is long-term tobacco use [1, 3]. Long-term exposure to asbestos dust is a further risk factor, mostly associated with occupational exposure e.g. in skilled trades and manufacturing industries, such as demolition, renovation and maintenance [1, 3–6]. As tobacco consumption and exposure to asbestos dust often covary and possibly have synergistic effects in cancer causation, there is an even greater risk of developing lung cancer in those individuals [7].

As lung cancer often is asymptomatic in early stages, it is predominantly detected in advanced stages [1, 8]. In these stages a cure and rehabilitation are often hard to achieve. and patients must undergo more invasive and stressful therapies. To detect lung cancer in early stages and hence reduce morbidity and mortality, screening with low dose computed tomography (LDCT) showed to be an effective method [9]. Individuals with a history of smoking who underwent asymptomatic LDCT screening showed an up to 20% reduced risk of dying from lung cancer in large randomized control trials in the United States [10] and the Netherlands [11]. The German Social Accident Insurance (DGUV) offers lung cancer screening to employees with a history of occupational asbestos exposure (EVA-LCS) [12]. By that, individuals are invited annually to take part in a medical consultation, during which they and their physician decide whether to undergo LDCT screening. This program’s distinctive criterion is that, in addition to smoking and age, occupational exposure to asbestos dust is the key inclusion criterion.

To evaluate and assess such cancer screening programs, it is important to understand who participates and whether certain groups of eligible individuals are underrepresented. Empirical studies of similar programs (e.g. LDCT screening for early lung cancer detection in smokers [13], colonoscopy for early detection of colorectal cancer [14, 15] and mammography for breast cancer [15]) discussed facilitators and barriers to participation. Possible facilitators included risk factors and disease-related symptoms, recommendation from healthcare professionals to participate, and the self-perceived effectiveness of screening. Potential barriers were older age, male gender and perceptions of masculinity, lack of knowledge and understanding of screening, low health literacy and fear of the screening method and procedure as well as concerns about being stigmatized due to behavioral risk factors [13–15].

However, the results of many predictors were inconclusive as to whether they were barriers or facilitators [15, 16]. Similarly, the relevance of these predictors remains unclear in the context of lung cancer screening (LCS) for individuals with occupational exposure to asbestos dust. This is because there are only a few studies on LCS for individuals exposed to asbestos dust. These studies are also of heterogeneous quality and fewer in number than those based exclusively on smoking status and age [6].

### Research interest

Given the importance of early detection of lung cancer, this study aimed to improve understanding of participation patterns in LDCT screening among individuals with a history of occupational asbestos dust exposure. The results could provide new insights into the growing body of literature on this highly relevant health policy issue, as well as concrete indications for interventions to increase participation in the screening program (e.g. public health education targeted at specific groups), impulses for efficient health care and impetus for further research on the inclusion or exclusion of predictors. For clinical practice, the aim was to gain a better understanding of willingness to participate and, if necessary, to develop interventions to increase participation.

## Methods

### Study design and data basis

This study was conducted within the scope of the EVALUNG project, which evaluates EVA-LCS using quantitative and qualitative methods [17]. Ethical approval for the quantitative work packages using anonymised routine data was obtained from the Ethics Committee of the Hamburg Medical Association on the 14th of July 2023 (reference number 2022-100917-BO-ff).

Since 2014, the DGUV offers EVA-LCS to insured individuals with occupational asbestos dust exposure. Inclusion criteria for the main cohort are ≥ 10 years of occupational asbestos dust exposure with onset before 1985, ≥ 30 pack years tobacco consumption and age ≥ 55 years [12]. Eligible individuals of the main cohort are invited annually (including reminders where necessary) to undergo a scheduled medical consultation followed by an optional LDCT scan. Further information regarding EVA-LCS can be found in Duell et al. 2022 [12] and the study protocol for EVALUNG [17].

The selected time frame for this study’s data collection was 2014 to 2024. The individuals included in this study were those of the main cohort who met the eligibility criteria mentioned above and had their initial medical consultation in EVA-LCS. The aim of this cross-sectional analysis of routine data was to determine (1) the proportion and demographics of eligible individuals and participants in the initial medical consultation; and (2) the proportion and predictors of subsequent participation in LDCT screening. An exploratory statistical model was developed to identify and evaluate specific related sociodemographic risk factors and health-related predictors that increase the likelihood of participating in LDCT screening following the medical consultation.

### Data analysis

Descriptive statistics (absolute and relative frequencies, measure of central tendency and variability, histograms) were used on the one hand, and inferential statistics (binary logistic regression analysis) on the other hand.

The statistical requirements for the logistic regression model (binary outcome, linearity assumption of the predictors, independence of the predictors, absence of outliers, absence of multicollinearity among the predictors [18]) were checked in advance. To evaluate the binary logistic regression model, it was tested for statistical significance (*p* < 0.05) and the goodness of fit was tested using the Hosmer-Lemeshow-test (*p* ≥ 0.05; this test is designed to have a non-significant p-value to be rated as good [19]). Nagelkerkes R^2^ (theoretical values from 0 to 1) was used to interpret the explanatory power of the overall model. Values above 0.13% were rated as good [20]. Linearity of predictors was tested with Box-Tidwell test (as with Hosmer-Lemeshow-test, statistically non-significant values were rated as good [21]). To evaluate the individual predictors, statistical significance was tested (*p* < 0.05) and odds ratios (ORs) were employed. Multicollinearity was assessed using variance inflation factors (VIFs) (values < 5 were considered acceptable [22]). Cases with missing data were excluded from the regression analysis.

Further information regarding the study design, data collection and analysis can be found in the S1 Appendix (RECORD-Guideline [23]), the S2 Appendix (Data Processing) and the study protocol [17].

## Results

### Cohort and descriptive results

From 2014 to mid-2024, a total of 26,353 individuals from the main cohort were considered eligible to participate in EVA-LCS. 16,822 individuals (63.8%) underwent at least the initial medical consultation in EVA-LCS. Of these, 14,491 (55.0%) persons met this study’s inclusion criteria, from now on called “study participants”. 10,714 (73.9%) of the study participants agreed to have a subsequent LDCT screening. Further information can be found in the S3 Appendix (Flow Diagram).

The cohort of eligible individuals was predominantly male (97.2%). Their mean age was 67.4 (median 67, SD 7,9) at the time of their enrolment into EVA-LCS. Eligible individuals’ most common (former) field of professional activity was insulation (Table 1). Overall, there were no noticeable differences between the characteristics of eligible individuals and participants of the medical consultation. However, the results indicate that the cohort of participants was younger than the cohort of eligible individuals (Table 1), particularly when accounting for the time lag between enrolment and the initial medical examination. Mean age at medical consultation was 66,9 (median 66, SD 7,2).

**Table 1:** Eligible individuals’ (*n* = 26,353) and study participants’ (*n* = 14,491) characteristics

	Eligible individuals ^a^ (*n* = 26,353)		Study participants ^b^ (*n* = 14,491; 55.0%)	
	n	%	n	%
Sex				
Female	735	2.8	375	2.6
Male	25,618	97.2	14.116	97.4
Age group (years) ^c^				
55–65	11.964	45,4	6,828	47.1
66–75	9.669	36.7	5,630	38.9
76–85	4.346	16.5	1,945	13.4
>85	357	1.4	84	0.6
(Former) field of professional activity ^d^				
Insulation (e.g. thermal insulation, sound insulation, fire protection)	4,607	19.3	2,566	19.7
Maintenance work (e.g. turbines, furnaces, pipes)	2,895	12.2	1,600	12.3
Vehicle repair, brake and clutch service	1,972	8.3	1,161	8.9
Other ^e^	11,318	47.5	6,043	46.4
Unknown	3,023	12.7	1,662	12.8
States ^f^				
Baden-Wuerttemberg	1,344	5.1	643	4.4
Bavaria	2,214	8.4	1,181	8.2
Berlin	987	3.7	554	3.8
Brandenburg	1,232	4.7	673	4.6
Bremen	490	1.9	313	2.2
Hamburg	643	2.4	385	2.7
Hesse	1,342	5.1	768	5.3
Lower Saxony	2,489	9.5	1,529	10.6
Mecklenburg-Western Pomerania	688	2.6	353	2.4
North Rhine-Westphalia	7,286	27.7	3,924	27.1
Rhineland-Palatinate	1,365	5.2	743	5.1
Saarland	327	1.2	176	1.2
Schleswig-Holstein	924	3.5	538	3.7
Saxony	2,004	7.6	1,110	7.7
Saxony-Anhalt	2,379	9.0	1,282	8.8
Thuringia	622	2.4	316	2.2

^a^ Data reported at enrolment into EVA-LCS. ^b^ Only cases that met the inclusion criteria and had their initial medical consultation were considered. ^c^ *n* = 17 missing for ‘Eligible individuals’ ; n = 4 missing for ‘Study participants’. ^d^ n = 2,538 missing for ‘Eligible individuals’; n = 1,459 missing for ‘Study participants’. ^e^ The rest was summarized from categories that comprise < 5% of all cases. ^f^ n = 17 missing for ‘Eligible individuals’; n = 3 missing for ‘Participants’

Study participants reported an average exposure to asbestos dust of 21.2 calendar years (median 20, SD 9.3) and 43.0 pack years of cumulative tobacco consumption on average (median 40, SD 12.1) at their initial medical consultation, 42.6% were active smokers.

The prevalence of pre-existing pulmonary conditions, as assessed by the physician in the structured questionnaire, ranged from 1.6% to 16.1% at the time of their medical consultation. The most common condition was pneumonia in the past. Current pulmonary symptoms varied between 0.5% and 16.6%. The most common symptom was a cough (Table 2).

**Table 2:** Participants’ pre-existing pulmonary conditions and current symptoms at the time of medical consultation (*n* = 14,491)

	*n*	%
Pre-existing pulmonary conditions		
Pulmonary tuberculosis ^a^	230	1.6%
Pneumonia ^b^	2,330	16.1%
Pleuritis ^c^	798	5.5%
Bronchitis	1,646	11.4%
Asthma	730	5.0%
Current pulmonary symptoms		
Cough	2,402	16.6%
Sputum	2,061	14.2%
Hemoptysis	76	0.5%
Hoarseness	773	5.3%

^a^ *n* = 131 not included (“unknown”), ^b^ *n* = 245 not included (“unknown”), ^c^ *n* = 244 not included (“unknown”)

Out of the *n* = 14,491 study participants that had their initial medical consultation, *n* = 10,714 (73.9%) underwent subsequent LDCT screening of the lungs.

### Regression analysis

A binary logistic regression model with 13 explanatory variables and “having a LDCT scan” as outcome was established to test the predictive character of the described variables (Table 3). The assumptions for the statistical model were tested: the independence of the sample and the binary coding of the outcome variable were given. The linearity of the presumed correlation with participation in an LDCT screening for metric variables were given for age (*p* = 0.981) and years of asbestos exposure (*p* = 0.368). Pack years was removed from the overall model, since its linearity assumption seemed too close to fail (*p* = 0.060) and was replaced by smoking status (binary: smoker vs. former smoker).

The overall model included *n* = 14,020 study participants, was statistically significant (*p* < 0.001) and had an adequate goodness of fit (Hosmer-Lemeshow-Test *p* = 0.276). The total variance explained (Nagelkerkes R^2^) was 0.026 and thus rated as low. The VIFs varied between the single predictors from 1.0 to 2.5; multicollinearity was therefore considered negligible. Cook’s distance values were < 0.1 and outliers were therefore considered acceptable.

73.9% of the regression sample underwent an LDCT scan of the lungs (null-model prevalence OR: 2.8). Three out of the 13 predictors showed a statistically significant association with undergoing an LDCT scan: Younger participants (OR: 0.96, *p* < 0.001), participants with a longer period of occupational exposure to asbestos dust (OR: 1.01, *p* = 0.011) and participants with pre-existing pneumonia (OR: 1.16, *p* = 0.011).

**Table 3:** Binary-logistic regression model (n = 14,020)

Outcome variable: participation in LDCT screening of the lungs (0 = no, 1 = yes)			95% CI		
*R*^2^ = 0.026	B	OR	LL	UL	p
ρSociodemographic					
Sex (female vs. male)	0.11	1.12	0.88	1.42	0.352
Age (years)	-0.04	0.96	0.95	0.97	< 0.001*
Risk factors					
Asbestos exposure (years)	0.01	1.01	1.00	1.01	0.011*
Smoking status (smoker vs. former smoker)	-0.03	0.97	0.89	1.05	0.432
Current pulmonary symptoms					
Cough	-0.07	0.94	0.79	1.10	0.416
Sputum	0.12	1.12	0.95	1.34	0.185
Hemoptysis	-0.44	0.64	0.39	1.06	0.081
Hoarseness	0.10	1.11	0.93	1.32	0.260
Pre-existing pulmonary conditions					
Pulmonary tuberculosis	0.16	1.18	0.86	1.60	0.307
Pneumonia	0.15	1.16	1.03	1.29	0.011*
Pleuritis	-0.06	0.95	0.80	1.12	0.518
Bronchitis	0.05	1.05	0.91	1.20	0.517
Asthma	0.10	1.10	0.92	1.33	0.304

B = unstandardized beta, CI = confidence interval, LL = lower level, UL = upper level, OR = odds ratio,* statistically significant (p< 0.05)

## Discussion

This study examined individuals with long term occupational asbestos dust exposure and a smoking history of at least 30 pack years, to determine which predictors increase the probability to have an LDCT scan of the lungs following a medical consultation. About 74% of participants that met this study’s inclusion criteria in EVA-LCS underwent an LDCT scan. Overall, a statistical model was developed that was able to identify predictors that have a systematic but also weak association with participation in LDCT screening. The analyses do not allow us to understand the actual decision-making process between eligible individuals and physicians for having or declining an LDCT scan.

### Participation rate

Our raw participation rate in the medical consulting (63.8%) is higher compared to other studies with a similar research focus (52.3% participation in lung health check appointments prior to LDCT scan in the Lung Screen Uptake Trial [24] and 50.8% for a telephone-based risk assessment in the Yorkshire Lung Screening Trial [25]; 53.0% participation in an eligibility assessment prior to LDCT scan in the study of Bhamani et al. [26]). In the DEP’KP80 trial, 75.0% of individuals invited by their physician underwent a baseline LDCT scan [27], which is comparable to the results (73.9%) of our study. Crosbie et al. reported a participation rate of 84.7% in LDCT scan after telephone triage [25]. Organizational structures that include invitation strategies or algorithms with timed appointments and reminders, as used by Bhamani et al., Crosbie et al., Quaife et al. and in EVA-LCS, are beneficial for a higher participation rate in LCS [24–26]. Bhamani et al. found that, compared to an open invitation, inviting individuals with a timed appointment was the main predictor of response to an initial invitation (OR: 2.66, *p* < 0.001) [26]. Couraud et al. reported that forgetting about the appointment or schedule issues were self-reported reasons for non-participation in LCS [27].

Our study deviates from the cited studies regarding sample population (e.g. participants’ age and smoking status) and study design (e.g. invitation method and organizational structure of the screening program, intervention, research setting). None of the cited studies included asbestos-related eligibility criteria. Our results therefore complement the growing body of existing literature on this topic, suggesting that individuals with a history of occupational asbestos exposure have a comparable participation rate in LCS to other eligible individuals.

### Predictors

#### Age

In our study, younger participants were more likely to undergo a LDCT screening following the medical consultation. Therefore, our results add to the existing mixed outcomes regarding the predictive character of age in LCS [16] and other preventive care programs (e.g. colonoscopies) [15, 28]. Our results may be explained by the possibility that physicians might no longer perceive the health benefits of LCS for older individuals [29, 30]. This might be an important aspect, as healthcare professionals’ recommendation plays a key role in individuals’ consent in screening programs [31]. The literature has also reported practical barriers to participation in healthcare services, such as travel and transport [31]. It can be assumed that these barriers mainly apply to older individuals. To better understand the predictor ‘age’ in relation to facilitators and barriers in LCS for individuals exposed to asbestos dust, this should be tested for interaction effects in future studies, e.g. with practical barriers and healthcare professionals’ recommendations.

#### Risk factors

Our study showed that individuals’ smoking status (current vs. former smoker) had no influence on participation. However, duration of occupational exposure to asbestos dust was found to be statistically significantly associated with participation. Pack-years were not used as a predictor since the linearity assumption was not met.

A reason for the statistically significant association between duration of occupational exposure to asbestos in our study could be the conceptual structure of this specific and targeted screening program by the DGUV and the resulting implications for medical consultation: EVA-LCS focuses on individuals with long-term asbestos dust exposure [12, 17]. It can therefore be assumed that the risk factor of asbestos exposure played a more important role than smoking status in the medical consultation and the resulting decision regarding LDCT screening. Also, having (former) colleagues diagnosed with lung cancer, which is positively correlated with amount of occupational asbestos exposure, might have led to increased participation rate [13]. On the other hand, judgement of current smoking status by physicians is a known barrier to utilization of LCS [13].

#### Pre-existing pulmonary conditions and symptoms

Our data showed that participants who have suffered from pneumonia in the past are more likely to undergo LDCT screening of the lungs following the medical consultation. Other pre-existing pulmonary conditions or current pulmonary symptoms assessed in the medical consultation did not show any statistically significant associations. This could be due to methodological reasons, as pneumonia has a comparatively high prevalence and thus variability in our data set. However, we had expected more pre-existing conditions and symptoms to be statistically significantly associated with LDCT screening participation, given that the literature suggested disease-related conditions and symptoms encourage participation [15, 16, 31]. Research indicates that anxiety represents a significant barrier to the uptake of LCS [13]. Since a lot of patients who have previously suffered from pneumonia presumably have already undergone LDCT scans before, this prior experience could contribute to reduced worry regarding LCS. Suggested higher risk of lung cancer in patients with previous pneumonia might have led to a stronger recommendation for participation by the physician [32].

#### Discussion of non-significant predictors

The symptoms are particularly noteworthy among the statistically non-significant predictors: by definition, screening programs are aimed at asymptomatic, apparently healthy individuals [33, 34]. Therefore, it was not expected that the symptom-related variables would have been statistically significant with positive correlation or had high explanatory power. However, it is interesting that they did not show a negative correlation, given that the presence of unique lung cancer symptoms in risk groups could lead to diagnostic clarification and therefore act as a barrier to participation in EVA-LCS, which aims at asymptomatic individuals. Another reason for symptom-related null results might be that individuals with severe symptoms (e.g. hemoptysis) are already being treated outside of EVA-LCS in the context of diagnostic evaluation and are therefore not participating in EVA-LCS. Also, participants who report hemoptysis during a medical consultation are likely to be referred to an external facility outside of the context of EVA-LCS for timely diagnostic evaluation. Therefore, our results regarding symptoms and pre-existing conditions could be slightly biased, as these participants might undergo an LDCT scan outside of EVA-LCS and this is not documented in our data. Overall, the null results suggest that symptoms reported by participants may be handled inconsistently in medical consultations regarding the subsequent LDCT screening. Another methodological reason for our symptom-related null results might have been a low prevalence in symptoms (e.g. hemoptysis), as this imbalance is a source of bias in logistic models [35].

The existence of disease-specific risk behavior for participation in corresponding screening programs is discussed controversially in the literature [16]. Previous findings suggest that the prevalence of disease-related risk factors increase participation in LCS [16] and other health services [15]. Though, other studies found that current smokers are less likely to participate in LCS compared to former smokers [24–26]. Current or past smoking were associated with perceived stigma or feelings of shame and were therefore barriers in participating in LCS [13, 31]. It should also be noted that we were unable to use pack years as a predictor in the model due to failed linearity assumptions, so we resorted to using current smoking status instead. Information on the duration of smoking cessation was also lacking. Our null result regarding smoking status as a predictor contributes to the existing body of literature on this topic, which contains mixed findings.

### Strengths and limitations

A key strength of this study is its use of a full sample of eligible individuals to present empirical data on participants’ decision-making, a topic of high relevance to cancer screening programs. The significance of suspected predictors could be evaluated, thereby contributing to the literature.

However, this study also had limitations: the data used is administrative routine data that was not primarily collected for research purposes. Hence, the selection of variables, data quality, and investigation of specific hypotheses have been very limited [36] – also due inconsistent documentation, possibly even more in the pilot phase of EVA-LCS. The conclusions that can be drawn based on health-related predictors (pulmonary and non-pulmonary) are limited. Another methodological limitation is the empirical imbalance in some risk factors (e.g. symptoms and pre-existing conditions) and sociodemographics (female gender), which might have slightly biased the statistical results. As many details are only documented during the medical consultation, we were unable to include eligible non-participants (meaning those who decide to opt out of EVA-LCS before this consultation) in the analysis. Consequently, our sample of participants may be biased towards healthier individuals and those with a more positive attitude towards prevention and early detection.

### Clinical implications

The small effects identified and the null results for symptoms indicate that the extent to which individual decision-making can be predicted using the routine data from this study is limited. It may be that the participants’ characteristics are less relevant than the specific consultation situation and the physician’s recommendation, which is one of the main facilitators of participation in LCS, as reported in the literature [13], even among individuals who perceive their own risk as low [37]. Additionally, the results point to the high relevance of clarification of risk factors for lung cancer in the population. Apparently, participants of EVA-LCS are aware of the risk of asbestos exposure, maybe because e.g. former colleagues suffered from asbestos related diseases or the communication of the asbestos ban. Though, the greater risk factor, (current) smoking status, did not significantly influence decision making. Good risk-related communication by physicians during medical consultations is therefore important.

Our results regarding the participation rate suggest that scheduled appointments facilitate participation in LCS and should be considered in future research and screening programs.

### Future research

The exploratory results of this study have implications for further research. On the one hand, the model should be expanded to include the additional predictors discussed (e.g. non-pulmonary comorbidities) to improve the decision for or against LDCT screening and test for possible interaction effects. To gain a more detailed understanding of participation in EVA-LCS, future studies should survey individuals who are eligible for LCS but decide not to take part in the preceding medical consultation. This could substantiate the conclusion regarding the clinical implications of this study. As part of the EVALUNG project, qualitative interviews with participants and physicians were conducted to gain a deeper understanding of this decision-making process. The results could serve as a basis for the inclusion of additional quantitative predictors. Furthermore, the results of the LCS, including detection rate and the distribution of tumor stages, will be presented and discussed in subsequent publication upon completion of the project.

## Conclusion

This study based on real-world routine data demonstrated which sociodemographic, risk-related and disease-related factors contribute to participation in LDCT screening of the lungs following a medical consultation and which factors are not relevant. The results provided strategies for concrete interventions and further research to encourage greater participation in screening programs for this highly relevant health policy issue among individuals with history of occupational exposure to asbestos dust.

## Supplementary information

Below is the link to the electronic supplementary material.

Supplementary Material 1

Supplementary Material 2

Supplementary Material 3

## Data Availability

In accordance with data protection regulations, the quantitative data have been provided by DGUV’s Preventive Healthcare Service on the condition that they will not be passed on to third parties. The datasets generated and analysed for EVALUNG are therefore not publicly available and must not be disclosed to external entities. Please contact the corresponding author for further details.

## Abbreviations

DGUV: German Social Accident Insurance
LCS: Lung cancer screening
LDCT: Low-dose computed tomography
OR: Odds ratios
SD: Standard deviation
VIF: Variance inflation factors
