# Supplementary material for: Who participates in LDCT screening for the early detection of lung cancer after long-term occupational exposure to asbestos dust? Results of the analysis of administrative and medical routine data from the German Social Accident Insurance
Source: BMC Pulm Med. 2026 Oct 12;26:461. doi: 10.1186/s12890-026-04780-4 (PMC13656686; doi:10.1186/s12890-026-04780-4)
Supplement: Supplementary file 2 [file 12890_2026_4780_MOESM2_ESM.pdf]

Appendix S2 (Data Processing)

Data for this study was anonymized by the DGUV's Preventive Healthcare Service prior to being provided to the research team (17). Table S2.1 presents a list of the utilized variables. The data was processed as follows: With the exception of gender and smoking status (current smoker vs. former smoker), non-binary variables were dichotomized for the regression analysis as follows: yes vs. no.

Further information regarding study design, case selection and data processing can be found in the S1 (RECORD-Guideline) and S3 (Flow Diagram) Appendix.

Table S2.1: Used data variables

Item	Values
Sex	female, male
Year of birth	4 digits
(Previous) occupation	internal 3-digit code (110-999)
State	Baden-Wuerttemberg, Bavaria, Berlin, Brandenburg, Bremen, Hamburg, Hesse, Lower Saxony, Mecklenburg-Western Pomerania, North Rhine-Westphalia, Rhineland-Palatinate, Saarland, Saxony, Saxony-Anhalt, Schleswig-Holstein, Thuringia
Date of initial medical consultation	DD.MM.YYY
Date of first LDCT screening	DD.MM.YYY
Asbestos dust exposure (calendar years)	2 digits
Cumulative tobacco consumption (pack years)	2 to 3 digits
Smoking status	current smoker, former smoker
Pre-existing pulmonary conditions<br>("Have you ever had any of the following illnesses?")	• pulmonary tuberculosis,<br>• pneumonia,<br>• pleuritis,<br>• bronchitis several times a year,<br>• asthma
Current pulmonary symptoms<br>("Have you experienced these symptoms?")	• cough for at least three months of the year,<br>• sputum for at least three months of the year,<br>• hemoptysis,<br>• persistent hoarseness, difficulty swallowing, or a sensation of having a lump in your throat recently

Data plausibility and missing data analysis were performed and are presented in Table S2.2. Overall, a small number of isolated implausible values were found (e.g. no documented pack years or years of asbestos exposure, 999 pack years), which is not uncommon in routine data. In accordance with the study's goals, we set the minimum years of asbestos exposure to 10 and pack years to 30. To decrease the impact of outliers, we set the maximum years of occupational asbestos exposure to 60 and pack years to 100. Values outside the specified range were designated as missing data. This set range resulted in 14,085 participants who met the study's inclusion criteria (see S3 Appendix).

Table S2.2: Missing data analysis and plausibility checks for the raw data of individuals in their first medical consultation (n=16,822)

	**Set range for study**	**Missing data**
**Quantitative Variables**		
Asbestos dust exposure (calendar years)	10-60	3.1%
Cumulative tobacco consumption (pack years)	30-100	2.7%
**Qualitative Variables**		
Smoking status	-	2,7%
Pre-existing pulmonary conditions	-	2,7%
Current pulmonary symptoms	-	2,7%
Data available for those participants only who underwent the medical examination		
