# Supplementary material for: Who participates in LDCT screening for the early detection of lung cancer after long-term occupational exposure to asbestos dust? Results of the analysis of administrative and medical routine data from the German Social Accident Insurance
Source: BMC Pulm Med. 2026 Oct 12;26:461. doi: 10.1186/s12890-026-04780-4 (PMC13656686; doi:10.1186/s12890-026-04780-4)
Supplement: Supplementary file 3 [file 12890_2026_4780_MOESM3_ESM.pdf]

Appendix S3 (Flow Diagram)

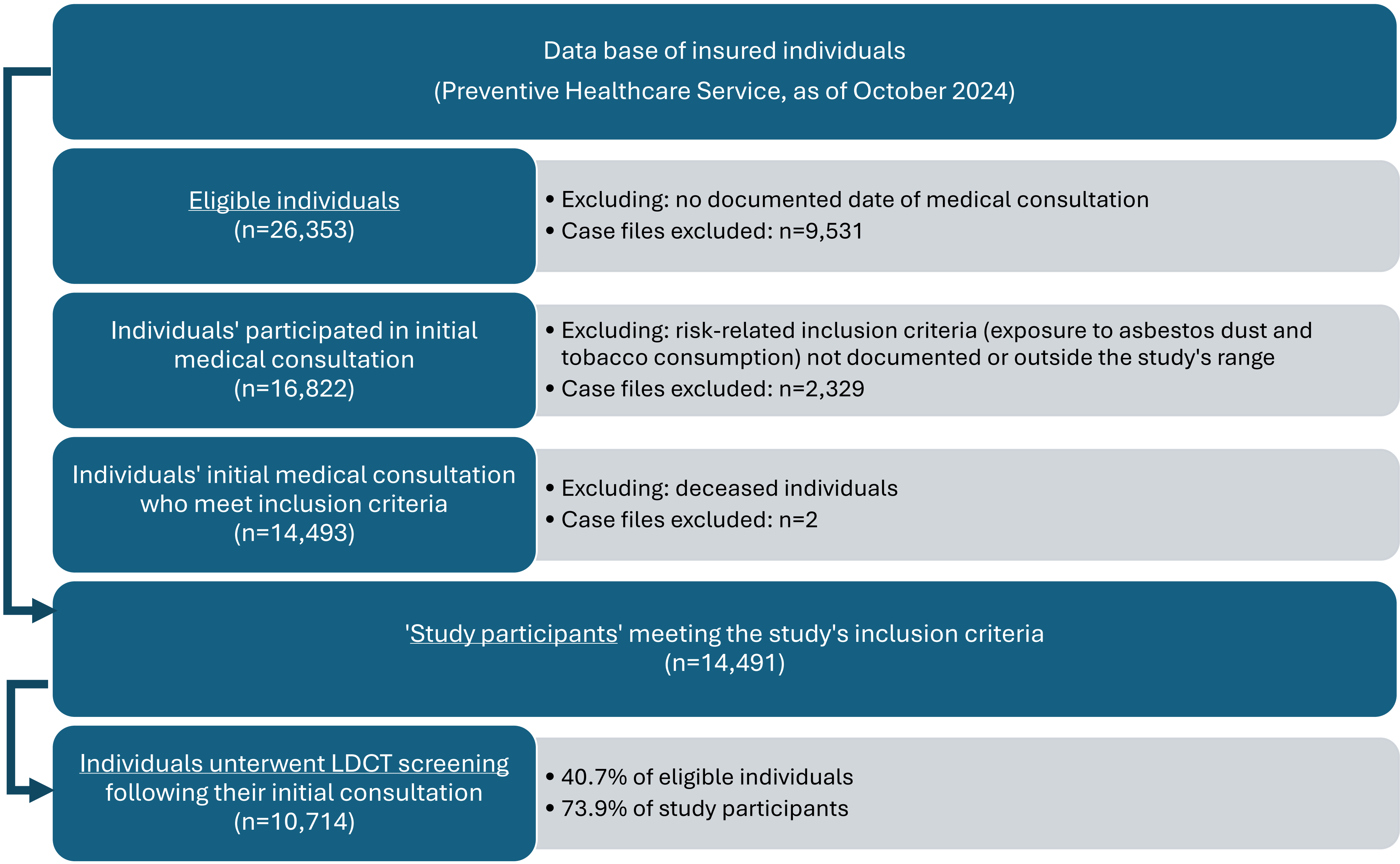

Oubaid, N. & Greiner, F. et al. Who participates in LDCT screening for the early detection of lung cancer after long-term occupational exposure to asbestos dust? Results of the analysis of administrative and medical routine data from the German Social Accident Insurance
